# Digital Twin for Centrifugal Extractors Exemplified for pDNA Clarification Process after Lysis

**DOI:** 10.1021/acsomega.4c04530

**Published:** 2024-07-05

**Authors:** Alexander Uhl, Axel Schmidt, Jochen Strube

**Affiliations:** Institute for Separation and Process Technology, Clausthal University of Technology, Clausthal-Zellerfeld 38678, Germany

## Abstract

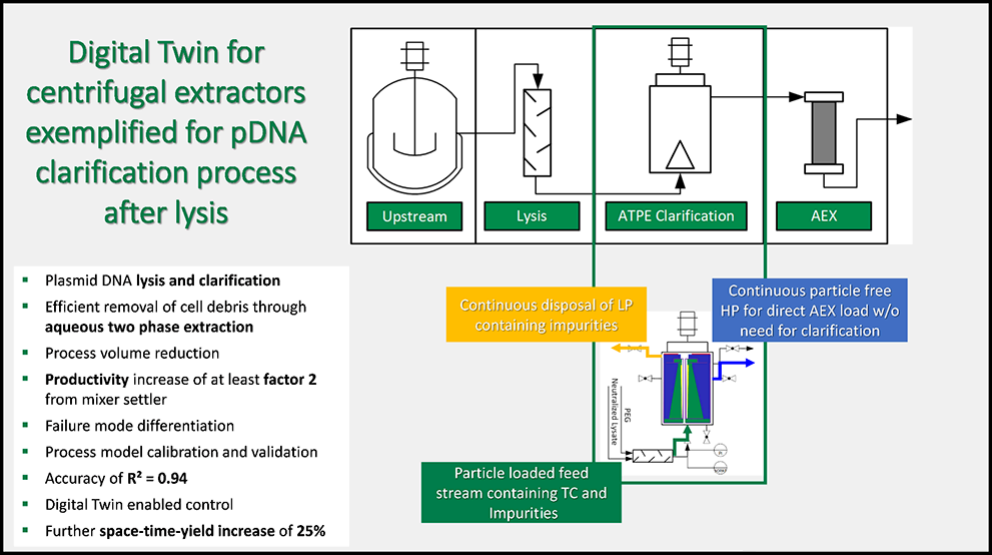

Plasmid DNA is an important substance for the pharmaceutical industry. A major challenge in its production is the clarification of the lysate after harvesting. In this work, a novel process for this is demonstrated in an annular centrifugal contactor (ACC) with an aqueous two-phase extraction. The ACC can increase the space-time yield of the clarification step by at least a factor of 2 compared to the horizontal continuous separator. A model for describing and predicting the conditions in the rotor is created for the ACC and calibrated with an accuracy of *R*^2^ = 0.94, which is sufficient for prediction in process design and operation. A digital twin (DT), which determines the purity of the phases at the outlet of the ACC, is calibrated with an accuracy of 95% for the light phase and 97% for the heavy phase, which defines in manufacturing operation the safety margin for both phases below the theoretical optimal operation point. By using the DT, an additional increase in productivity of 25% can be achieved in the ACC with model-predictive control.

## Introduction

### Plasmid DNA

Plasmid DNA (pDNA) is used as a therapeutic agent and for other biopharmaceutical applications. It can be used as a transfection agent for the production of vaccines and in its linearized form as a template for the production of mRNA vaccines. In particular, mRNA vaccines have been increasingly used in the immunization of the population in recent years.^1^

The production of large quantities of pDNA is achieved on an industrial scale by fermentation of *Escherichia coli* (*E. coli*) bacteria.^2^ After cell harvest, the downstream process starts with the plasmid recovery.^3^ This is done by chemical or mechanical destruction of the cell walls. The industrial standard for initial pDNA recovery is alkaline lysis.^4^ To prevent degradation of pDNA, a neutralization buffer is added subsequently. This can be done in batches in a continuously stirred reactor^5^ or in static mixers.^6^ Lysis also produces larger cell debris and denatured proteins, which must be removed from the process medium to enable a chromatographic purification step, and during neutralization, flocs are formed.^7^

Several options for clarifying the neutralizate have been published. One possibility is a combination of flotation and filtration with glass beads.^8,9^ Another is the use of a cross-flow filter cascade with three increasingly smaller filter cut-offs.^10^ Processes with density gradient depth filtration or membrane filters are also known.^11^

**Figure 1:**
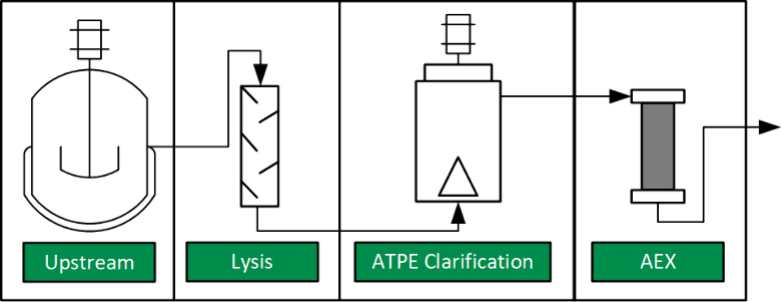
Schematic representation of pDNA manufacturing process.

Another approach to clarifying the neutralizate is the use of aqueous two-phase extraction (ATPE), whereby the neutralizate is brought into contact with a polymer-containing phase. This results in the formation of two aqueous phases. This has the advantage that the system does not stress the pDNA. By precisely adjusting the equilibrium, the process volume can also be reduced, which increases the productivity of the subsequent chromatographic purification. The cell debris is present in the interphase after the formation of the aqueous two-phase system (ATPS) and can be removed by continuous or discontinuous settling. This has already been shown for monoclonal antibodies and pDNA.^12,13^ The process is shown schematically in Figure 1.

### Centrifugal Extraction

Centrifugation accelerates settling and coalescence, therefore enabling shorter residence times in the apparatus. In addition, high mixing efficiency in the annular gap is achieved. Therefore, faster mass transfer and a lower mixer apparatus volume are required. This also means that a smaller footprint is necessary, i.e., area efficiency, a cost advantage for expensive GMP-approved production areas.

Stainless steel construction is very resistant to corrosive or caustic media and therefore easy to handle during CIP.^14^

**Figure 2:**
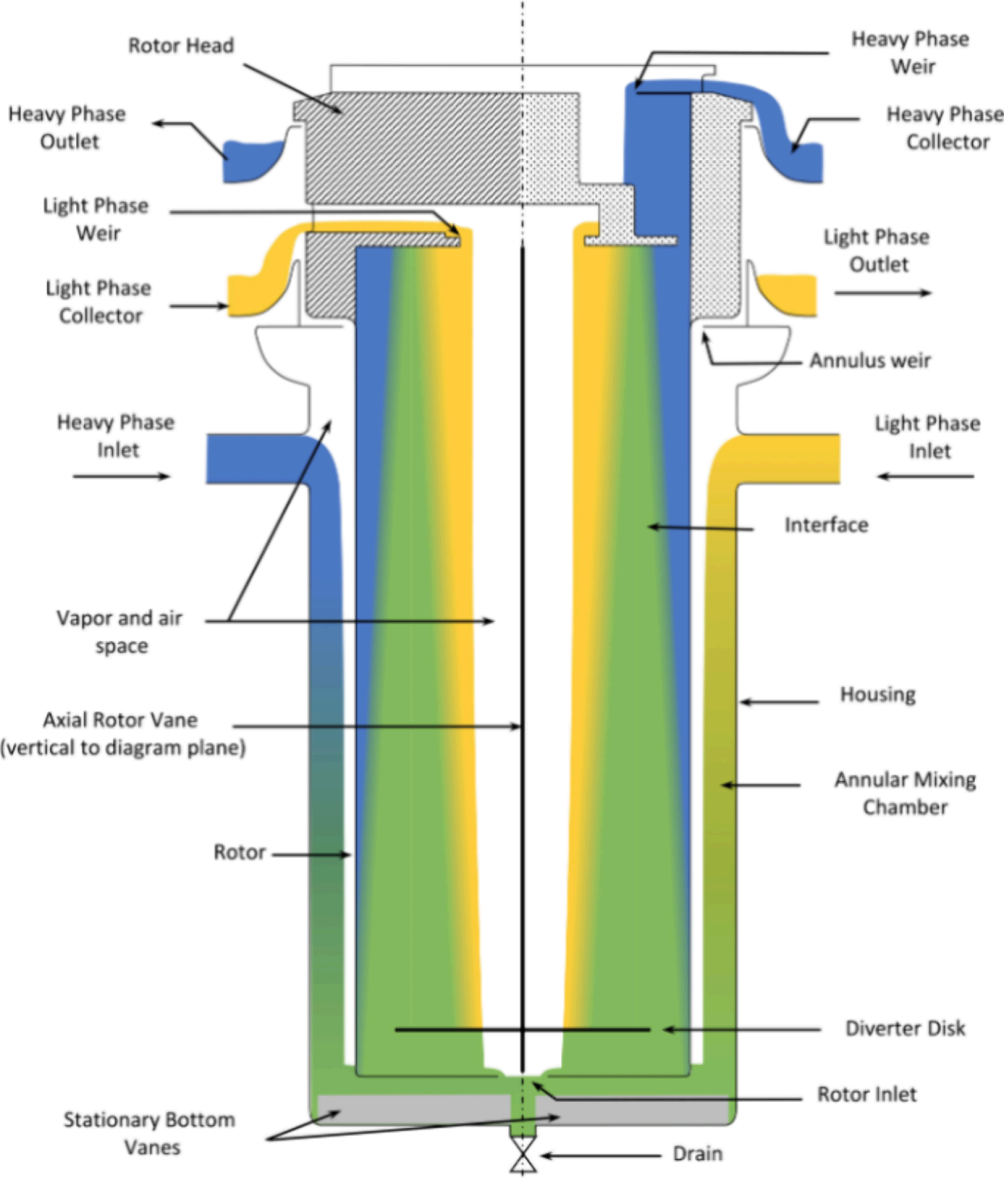
Schematic representation of the cross section of an ACC, reprinted from Hamamah and Grützner 2023.^16^

Figure 2 explains the schematic design of an annular centrifugal contactor (ACC), sometimes called a centrifugal extractor. The feed can enter tangentially to the rotor via the light and heavy phase inlets or axially from below through the drain. The blue color marks the heavy phase (HP), the yellow the light phase (LP), and the green the dispersion of both. The rotation of the inner cylinder (rotor) creates a dispersion in the annular gap, in which the droplet size depends on the rotational frequency.^15^ This flows into the rotor, where the centrifugal effect accelerates the settling of the phases. These hit the rotor head, where the light phase flows via the LP weir into the LP outlet. The heavy phase flows under the HP underflow, over the HP weir, and out of the HP outlet. The HP weir can be changed to influence the level of the phase limit. The selection is determined by a manufacturer-supplied macro based on the phase densities.

Centrifugal extractors have already been used successfully in the production of pharmaceuticals in the past. Penicillin was extracted from cell-free fermentation broth with *n*-butyl acetate or amyl acetate in several stages. The system tends to emulsify, so short extraction times are preferred, which are achievable by the ACC.^14,17,18^ The cell-free extraction of amino acid l-phenylalanine (product extract) with cell-free and protein-free fermentation broth by reactive extraction with kerosene/D2EPHA was found to be more efficient than the alternative with membrane extraction.^19^ Hydrocortisone was obtained from fermentation broth with a 6-step extraction in butyl acetate on an industrial scale. An ACC was used here, as the fermentation broth tends to emulsify with butyl acetate.^20^

These material systems represent cell-free purification steps. The centrifugal extractor can also be used to accelerate sedimentation by shutting off the outflow of the heavy phase and feeding through the bottom inlet to avoid crushing the particles in the annular gap, as in the clarification of rare earth element particles from phosphate acid.^21^

The aim of this work is to develop a robust clarification process for biomass-containing lysate for the subsequent production steps. For this purpose, a sufficient understanding of the process must be generated in order to understand and counteract the effects of deviations from the selected operating point. For this purpose, a model of the centrifugal extractor for the separation of an ATPE dispersion is created and calibrated with experimental data. In addition, the separation of the biomass from the lysate is demonstrated using the ACC.

The process development takes place within the framework of the Quality-by-Design (QbD) methodology.^22−27^ The critical quality attributes (CQA) of the product, in this case the purity of the heavy phase, are determined first. Further process attributes (PA) are the space-time yield. The influences of the process parameters on the CQA and PA are investigated experimentally by design of experiments (DoE) and modeling in order to develop a safe and optimized operating point. Robust operation point design is dedicated to 99.9% reliability. Furthermore, the developed and calibrated model can be used to predictively control the process as a digital twin (DT).

## Materials and Methods

### Materials

**Figure 3:**
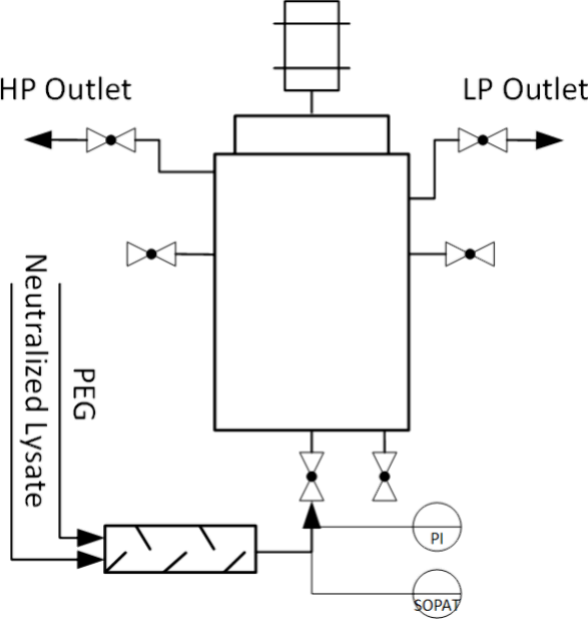
Setup for experimental work on the ACC.

The experiments are carried out on a CINC-V02 instrument (CINC Deutschland GmbH & Co. KG, Brakel, Germany) driven by a gear pump (Fisher Scientific, Hampton, NH, USA) and a Quattroflow pump (PSG Germany GmbH, Duisburg, Germany). The density is measured continuously, and the volume flow is measured by two mass flow sensors (Bronkhorst, Germany and Endress+Hauser, Germany). In order to minimize the influence of the shear mixing in the ring gap of the ACC of the phases, the inlet is selected via the bottom of the ACC. The phases are mixed using a static mixer (StaMixCo, New York, NY, USA). In previous work,^12,13^ this solution was found to be sufficient for the mass transfer of the ATPE. This has the advantage that the size of the droplets can be determined using a particle measurement probe (SOPAT GmbH, Berlin, Germany) before entering the ACC. Furthermore, the back pressure of the ACC is measured before the ACC (Autosen, AP016, Essen, Germany); see Figure 3.

The following buffers are used to prepare the lysate. The resuspension buffer P1 is made up of an aqueous solution of tris(hydroxymethyl)aminomethane hydrochloride (TrisHCl; VWR International, Radnor, PA, USA) and ethylenediaminetetraacetic acid (EDTA; AppliChem GmbH, Darmstadt, Germany). The lysis buffer (P2) is produced from sodium lauryl sulfate (SDS; Merck KGaA, Darmstadt, Germany) and sodium hydroxide (NaOH; Merck KGaA, Darmstadt, Germany). The neutralizing buffer (P3) is prepared from potassium citrate and citric acid (VWR International, Radnor PA, USA). For the ATPE, an aqueous polyethylene (PEG, Merck KGaA, Darmstadt, Germany) solution is prepared.

For the chromatographic analysis of the lysate, light and heavy phases of a weak ion exchanger (Tosoh Bioscience, Tokyo, Japan) are used with the mobile phase A of 20 mM TrisHCl at pH 9 and the mobile phase B of 20 mM TrisHCl and 1 M sodium chloride (NaCl; Merck KGaA, Darmstadt, Germany) at pH 9.^8^ Analysis is carried out with HPLC (Agilent Technologies, Santa Clara, CA, USA).

### Lysate Preparation and Analysis

The lysate is prepared by first resuspending wet cell paste (WCP) in the resuspension buffer for at least 1 h. P2 is added, and the mixture is stirred vigorously; lysis is stopped by adding cooled P3. ATPE is performed by adding the PEG solution to the produced neutralized lysate.^13,28^

For the analysis of the pDNA titer via ion exchange chromatography, an established method is used. A volume of 5 μL of the sample is injected, and elution is performed with a 5 min linear gradient with mobile phases A and B. The elution is monitored at a wavelength of 260 nm.^8^

### Methods: ACC

For the measurement of the liquid phase hold-up and the disperse phase hold-up, the following procedure is applied, which was used by Schuur and Hamamah:^16,29^

1. Start the ACC at the selected rotational frequency
2. Start the pumps
3. Ensure that steady state has been reached by taking multiple samples from the outlets and documenting the changes after four residence times have elapsed^29^
4. Sample from the outlets for phase purity
5. Stop the pumps, and after the discharge of the phases from the outlets has ceased, empty the annulus via the lower outlet
6. Stop the rotor and empty the apparatus, determining the total volume and the phase ratio

The dispersed phase hold-up (*ε*_rot_) denotes the phase ratio in the rotor. This is calculated in eq 1.
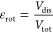
1*V*_dis_ is the volume of the disperse phase, and *V*_tot_ is the total volume or the liquid hold-up in the rotor.

### Model

The core of the developed model is a distributed plug flow (DPF) model. This can be divided into four parts: the accumulation, the convective and dispersive fluid dynamics, and a source or sink term. This model was already used by Uhl et al.^13^ to successfully describe and predict the horizontal continuous settler. The rotor of the ACC is discretized in the axial direction. The geometry of the rotor is simplified by simulating only one side, whereby the volume flow is adjusted so that the flow velocity corresponds to that expected from the measured total liquid hold-up in the rotor:
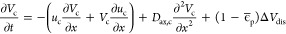
2
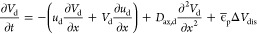
3
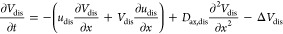
4where *V* is the volume of the respective phase in the discrete, *t* is the time, and *u* is the flow velocity in the respective phase. The coordinate of the discretization is represented by *x*. *D*_ax_ is the axial dispersion coefficient, and Δ*V*_dis_ the dispersion volume that coalesces in the discrete. This is calculated from a modified asymmetric film drainage model^30^ in eqs 5 and 7. The modification consists of replacing the earth acceleration in eq 5 with the acceleration in the centrifugal field (*a*) in eq 6.
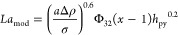
5
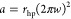
6
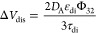
7*La*_mod_ represents the modified Laplace number, Δρ the density difference, σ the surface tension, *h*_py_ the height of the dispersion, and Φ_32_ the Sauter diameter of the dispersion, which is calculated analogously to previous works.^13^ The acceleration in the centrifugal field is made up of the rotational frequency *w* and the distance of the dispersion from the axis of rotation *r*_hp_. The coalescence time *τ*_di_ is calculated from *La*_mod_, which calculates the coalescence volume with the mean dispersion phase hold-up *ε*_di_ and half the diameter of the rotor *D*_A_.

The radius *r*_hp_ is calculated from the measured or modeled *ε*_rot_ by assuming complete settling. It can be imagined that both phases assume the geometry of a hollow cylinder, as the centrifugal force in the radial direction is much stronger than the gravitational force in the axial direction. This is a simplification for modeling. The height of the dispersion layer axially along the rotor can be simulated from the DPF model. From this information, the radius *r*_hp_ can be used to calculate where the inner and outer limits of the dispersion are located. If this is above the limit of the LP weir, the light phase will be impure; if it is below the HP underflow, the heavy phase will be impure.

The model parameter *ε*_rot_ is represented by an ordinary least-squares (OLS) regression and is dependent on the selected HP weir and the rotational frequency. The coalescence parameter *r*_s_^*^ and *ε*_dis__,0_ are determined analogously to previous works.^13,31^

## Results

### Experimental Results and Failure Modes

**Figure 4:**
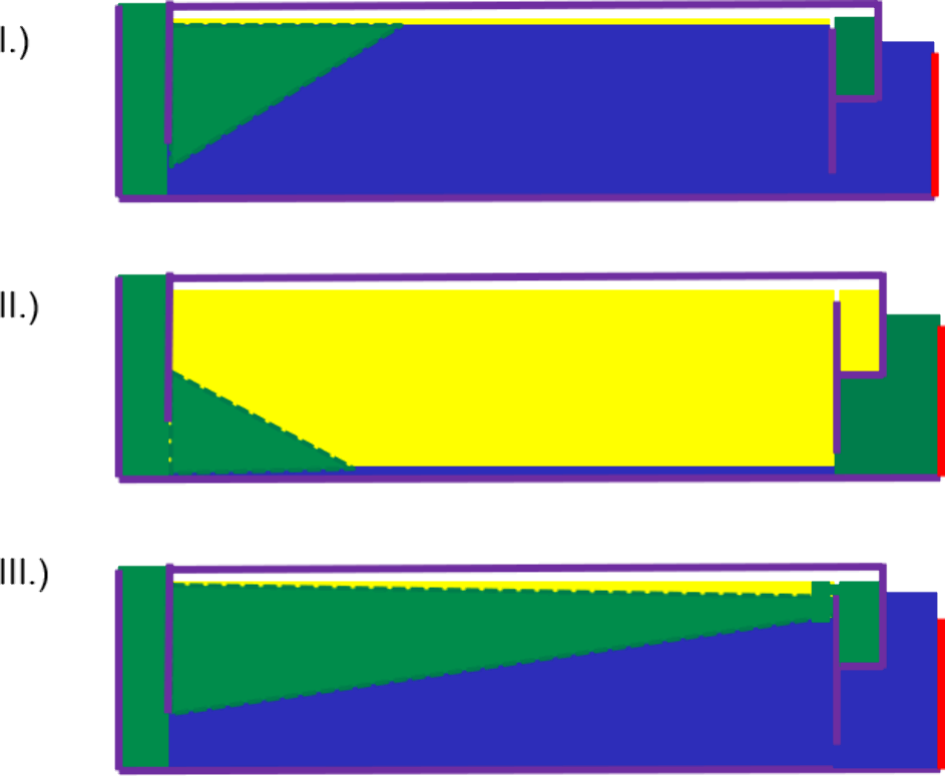
Failure modes of the phase separation in the ACC rotor, simplified representation, 90° rotated. Case I: impurity of LP outlet caused by high interface. Case II: impurity of HP outlet caused by low interface. Case III: impurity of LP outlet caused by insufficient coalescence.

For the evaluation, 85 results from a full factorial DoE with five different HP weirs (0.85–0.95), five different rotational frequencies (25–75 Hz), and three different flow rates (60–180 mL/min) are considered. In the initial DoE, the light phase with impurities is observed to leave the apparatus in six cases. This occurs at the HP weirs 0.85 and 0.875 up to the lowest speed of 25 Hz. A dispersion phase hold-up of over 0.94 is measured, which implies that, almost exclusively, the heavy phase is present in the rotor. The phase ratios in the rotor are shown in simplified form in Figure 4, Case I. In six further cases, it is observed that impure phases flow from the HP outlet. This occurs at the lowest HP weir 0.95 at the speeds 62.5 and 75 Hz; the lowest *ε*_rot_ of less than 0.24 is determined (Figure 4, Case II).

In addition, 10 experiments are carried out outside the initial test range. These are carried out with volume flows of up to 500 mL/min. Lower rotational speeds of 15 and 20 Hz are used with HP weirs of 0.925 and 1.0. Impurities of the light phase are observed for high volume flows of over 250 mL/min for 15 Hz and 1.0 weir. In addition, an *ε*_rot_ of 0.809 is measured on average. This implies that the cause of the impurity is not a too high or too low phase boundary level, but that the length of the rotor is not enough to completely coalesce the dispersion. Case III of Figure 4 occurs.

**Figure 5:**
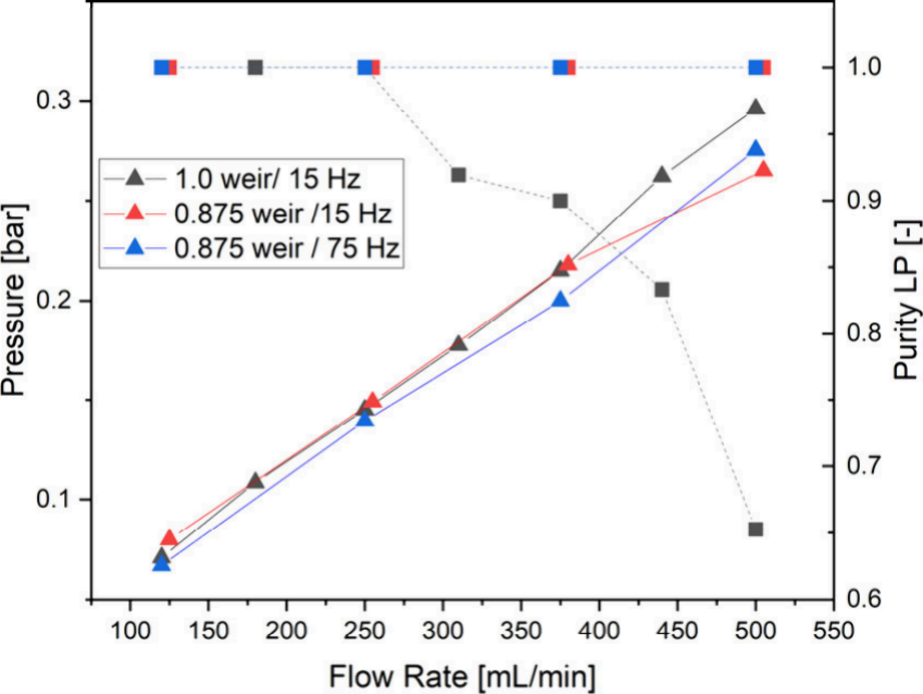
Illustration of the evaluation of some tests according to measured back pressure before the ACC (triangle) and purity of the phase from the LP outlet (rectangle).

The height or radius of the phase boundary in the rotor is calculated from the measured *ε*_rot_, assuming the cylindrical distribution of the heavy phase at the outer edge of the rotor and the complete separation of the dispersion. The limits of the complete separation of the phases are the height of the LP weir at 10.3 mm and the height of the HP underflow of 1.5 mm. The results of this are shown in Figure 5; the calculations are in good agreement with the observations from the experiments.

**Figure 6:**
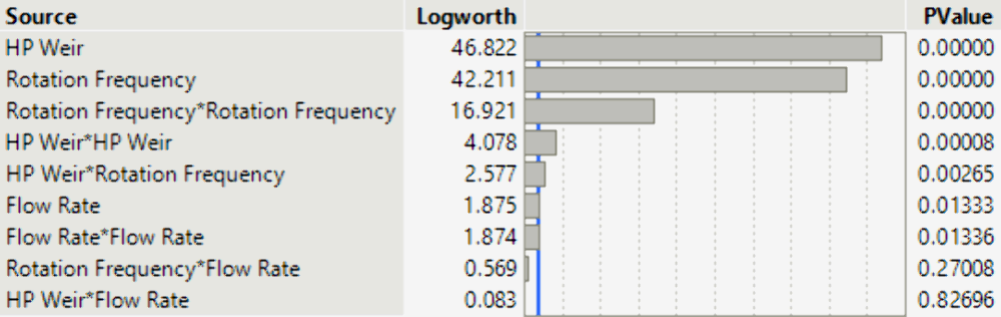
Statistical evaluation of the parameters on the dispersion phase hold-up in the rotor of the ACC.

Figure 5 shows the measured back pressure upstream of the ACC and the purity of the phases from the LP outlet. The back pressure is mainly dependent on the volume flow. The greatest restriction of the fluid flow in the ACC is in the path of the heavy phase. This could cause a buildup of the heavy phase caused by the dynamic pressure to change the phase boundary level to such an extent that the light phase leaves the rotor impure. When comparing the measured pressures and the purity of the light phase, it can be seen that impurity occurs when using the 1.0 weir and a rotational frequency of 15 Hz at a similar pressure at which pure phases can be obtained from the LP weir with other weirs and rotational frequencies. This shows that this phenomenon cannot be observed in the range of the tested parameters. This can also be proven by the statistical evaluation in Figure 6.

### Modeling Results

**Figure 7:**
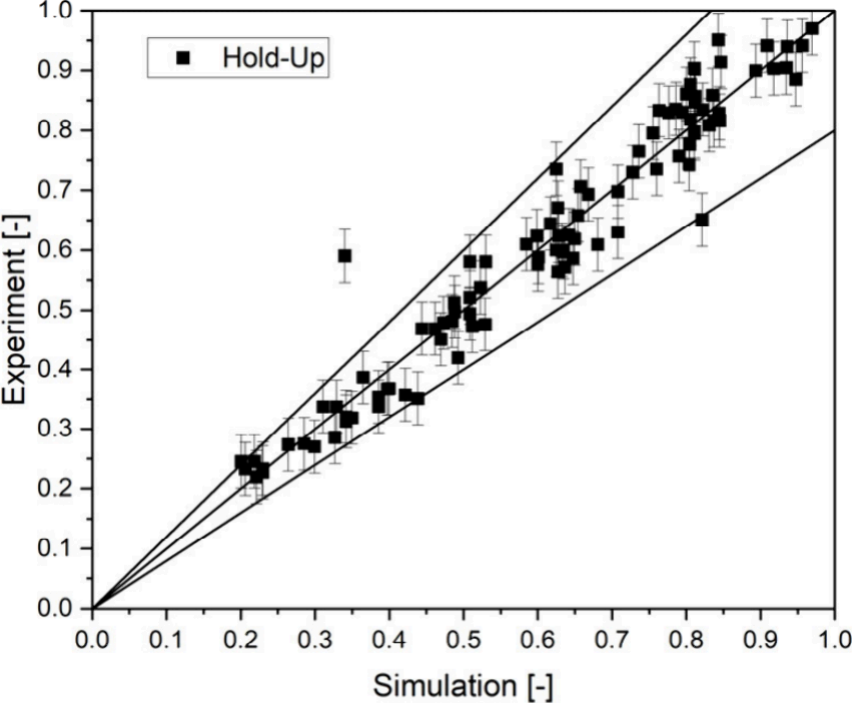
Model quality for the representation of the dispersion phase hold-up.

As can be seen from Figure 6, the dispersion phase hold-up in the rotor of the ACC is only dependent on the installed HP weir and the selected rotational frequency of the rotor. These results are used to form an OLS, which calculates the *ε*_rot_ from the HP weir and speed parameters. The results are shown in Figure 7. The error bars of the experimental values are determined from the triple determination of two experiments. The model has a quality of *R*^2^ of 0.94.

**Figure 8:**
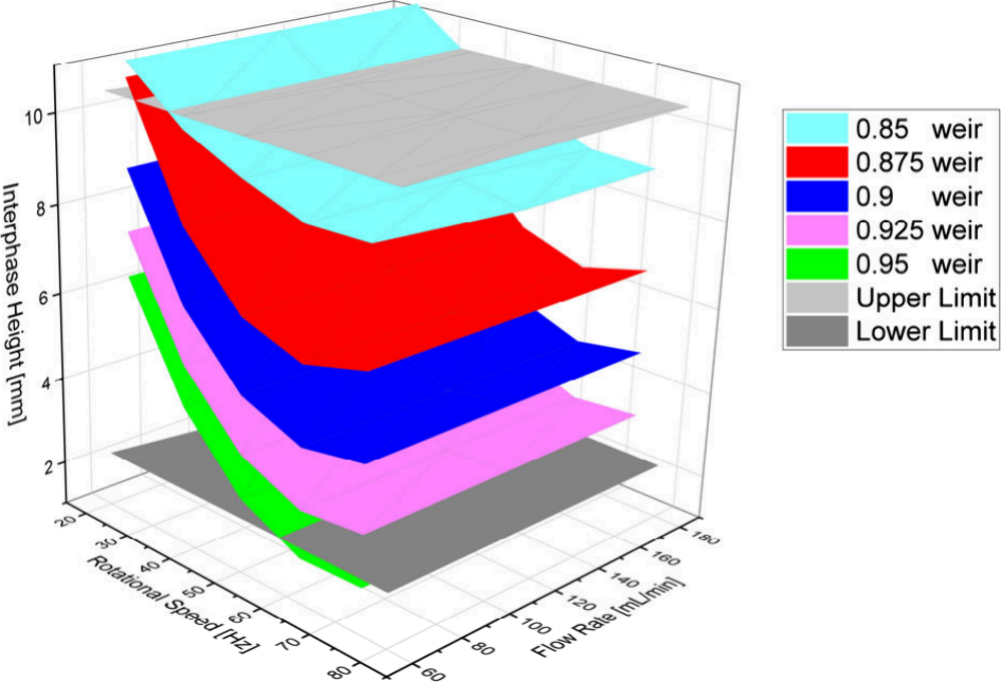
Level of the phase boundary from the calculated *ε*_rot_ of the tests.

As described, the level of the phase boundary can be calculated from *ε*_rot_. This also represents a correlation between the operating parameters and the first two failure modes from Figure 4. This relationship is given as a total overview in Figure 8.

**Figure 9:**
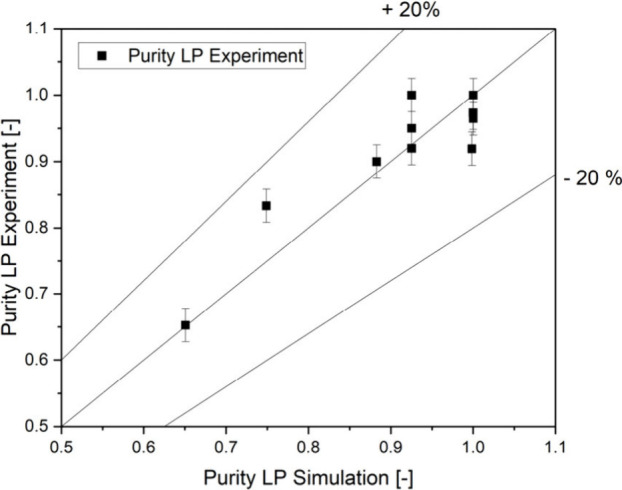
Quality of the calibration of the digital twin for the phase purity at the LP outlet.

This model for the dispersion phase hold-up in the rotor and thus the height of the phase boundary level is implemented in the coalescence model. The experiments are simulated with the created model, and a qualitative agreement is determined as to whether the HP or LP phase is impure. For the light phase, a match between the model and experiment is found in 95% of cases, and for the heavy phase, a match is found in 97% of cases. A quantitative determination of the purity could be carried out for the light phase. A coefficient of determination of *R*^2^ = 0.896 is achieved (shown in Figure 9). Since the aim is to ensure the purity of the phases and not to determine how impure they are, a high accuracy of the qualitative description of the purity is sufficient. The model can therefore be regarded as calibrated for ATPE.

### Failure Mode and Effect Analysis

**Figure 10:**
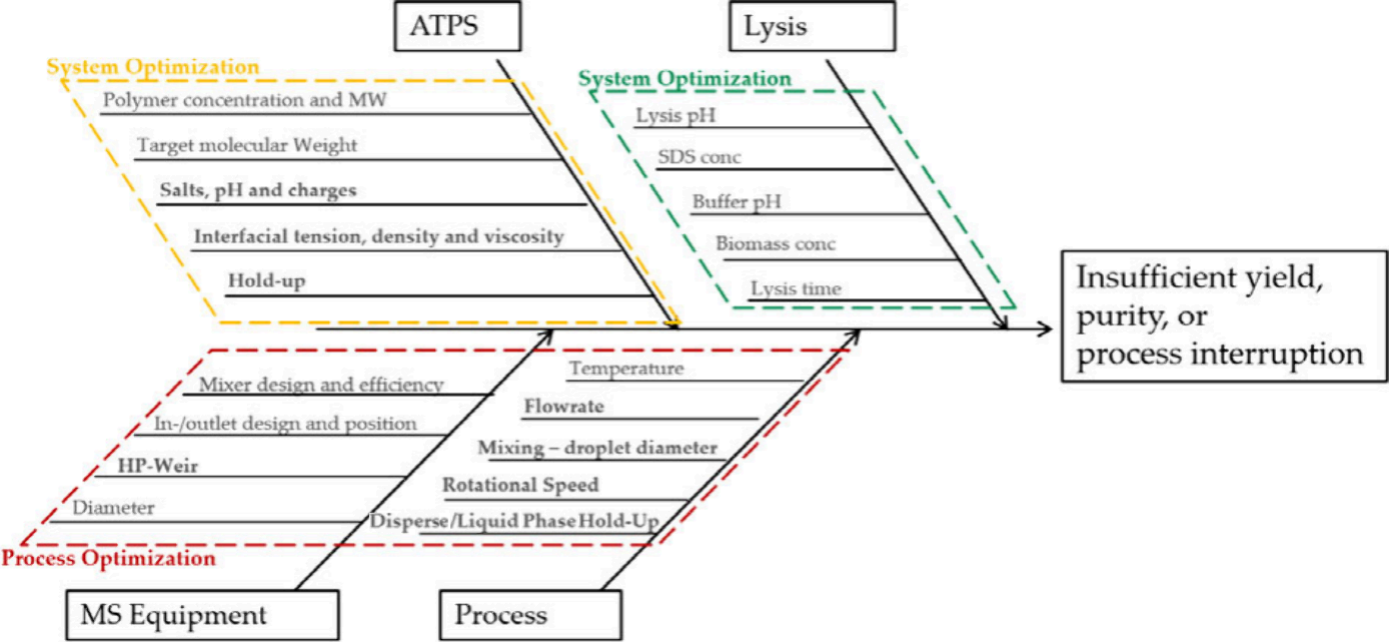
Ishikawa diagram showing the suspected and tested parameters for ATPE conducted in the ACC.

Figure 10 exemplifies the process parameters that can have an influence on the CQA and PA of the clarification of the lysate. These are divided into lysis, which has a particular influence on the concentration of pDNA, and ATPS, which has an influence on the material parameters density, viscosity, and surface tension due to the equilibrium. Furthermore, the process parameters consisting of the equipment-specific and process-specific parameters are considered.

Table 1 summarizes the absolute variations of the failure mode and effect analysis (FMEA) simulation study; these represent the maximum expected deviations from the selected operating point.

**Table 1:** Variation of the Important Parameters for the Sensitivity Study

parameter	variation
viscosity continuous phase	±5 mPa s
viscosity dispersed phase	±5 mPa s
density continuous phase	±25 kg/m^3^
density dispersed phase	±25 kg/m^3^
coalescence parameter	±0.05
dispersed phase hold-up	±0.1
initial droplet diameter	±1000 μm
flow rate	±250 mL/min
interfacial tension	±0.001 N/m
rotational frequency	±10 Hz

**Figure 11:**
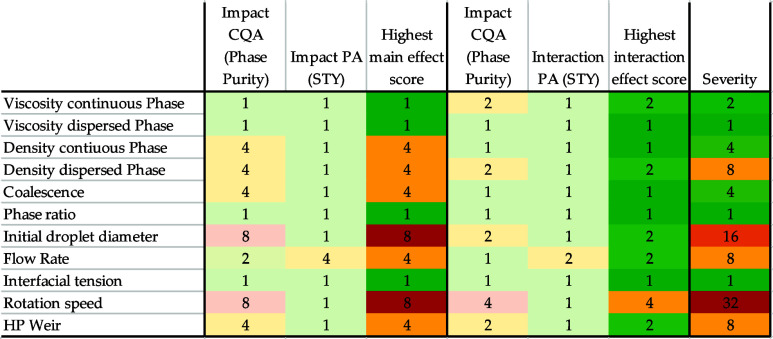
Qualitative conclusion of the sensitivity study. Highly sensitive parameters are marked in red, low sensitive parameters are marked in green.

To quantify the sensitivity of the parameters, 87 simulations are carried out. Initially, only variations of individual parameters and subsequently interactions of the parameters are investigated by a multifactor-at-a-time simulation study. The effects on the CQA of the phase purity and the PA of the space-time yield are shown as a qualitative evaluation in Figure 11. The most important parameters are the rotational vibration of the rotor and the initial droplet size of the dispersion. Furthermore, the density of the phases, the coalescence parameter, the volume flow, and the selected HP weir have a significant influence on the successful operation of the ACC.

### Extraction with Biomass

**Figure 12:**
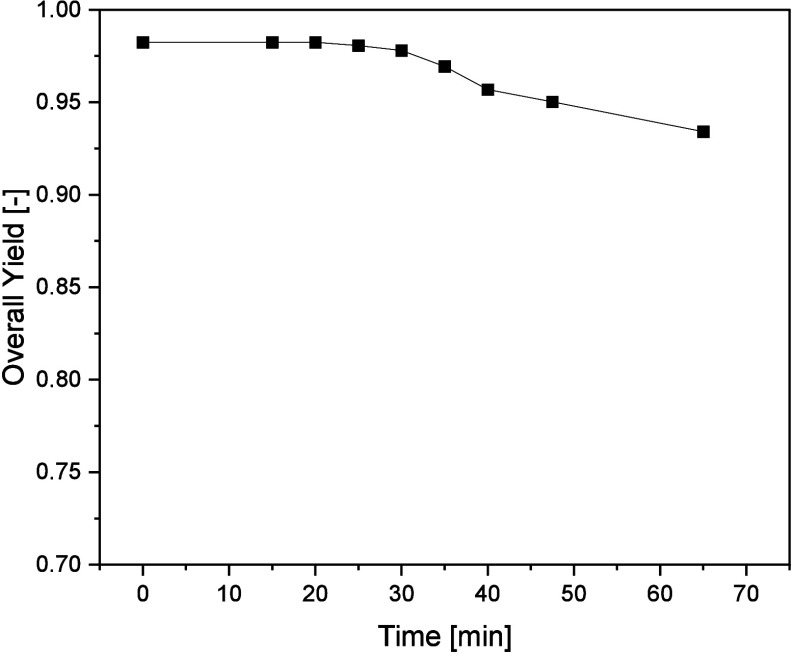
Overall yield of the biomass experiment over time.

Tested with wet cell paste (WCP), the operating point (HP weir and speed) is selected so that the phase limit is at the upper limit of the rotor. It is known from the previous experiments that the ATPS is only limited by coalescence above a volume flow of 1750 mL/min. Cell debris accumulates in the interphase between the light and heavy phases; the aim is to remove as much of the biomass out of the apparatus as possible with the light phase. The experiment ends after 65 min. Samples are taken from the LP and HP outlets at regular intervals in order to observe the purity and yield of the phases. This is visualized in Figure 12.

As is known from tests in continuous and discontinuous separators, a small part of the heavy phase adheres to the biomass, so a complete yield is not to be expected.^13^ It can be observed that the yield decreases slightly after 35 min, which can be attributed to the accumulation of biomass in the rotor. The accumulated biomass forms a dense cake in the apparatus, which cannot be rinsed out by the operation of the ACC. In volumetric terms, this biomass represents around 30% of the biomass from the entire batch. 50% of the biomass is measured in the collection tanks of the light phase. Thus, a reduction of biomass in the product phase of 80% is achieved over the entire process time; by the time the biomass breaks through after 40 min, the reduction in biomass is 94%. The yield of the heavy phase is 93.5% after the entire process time and 95.7% after 40 min.

With the chromatographic analysis, a titer for the neutralizate of 0.212 ± 0.005 g_pDNA_/L is measured; after performing ATPE, a titer of the heavy phase of 0.478 ± 0.023 g_pDNA_/L is determined. Within the light phase, the concentration of pDNA is 0.009 ± 0.001 g_pDNA_/L. An increase of plasmid DNA concentration by a factor of 2.25 is achieved with the ATPE as well as a yield of 97.0 ± 2.5%, excluding the HP loss through phase separation.

## Discussion

ATPE can facilitate the clarification of the process medium, as the biomass is not distributed in the product phase. By separating the phases, a large part of the biomass can be removed with the light phase. A challenge represents the fact that the biomass acts as an interphase in which the light and heavy phases are both present as well. This interphase can be compressed by the centrifugation, which leads to a gain in yield. ATPE is a gentle process for biologics that is also of use in productions other than pDNA.

In addition, the footprint of the apparatus used can be reduced by using the ACC. The CINC-V02 used here occupies an area of 30 × 30 cm, while a horizontal continuous separator with the same capacity for separating the biomass-laden ATPS occupies 60 × 30 cm. This means an increase in the space-time yield by a factor of 2. Dominantly, this comes into play even more when scaling up the process, as horizontal continuous separators take up proportionally much more area when the volume flow increases and ACC is scaled up mostly vertically. Nevertheless, a mixer settler has lower CAPEX, is simple to clean and maintain without moving parts like centrifugal devices, and is robust to operate.^12,13^

When operating the ACC, setting the phase boundary level is essential. This can be achieved by selecting the HP weir and the speed of the rotor. In this work, a statistical model with a high accuracy of *R*^2^ = 0.94 was developed to predict the phase margin level from the selected parameters. This can be used to develop an optimal operating point or to control a running process. Furthermore, this was linked to a rigorous coalescence model, which can predict the settling behavior and thus the phase purity with a high accuracy of 95% for the LP and 97% for the HP. This made it possible to calibrate a digital twin for this process appropriately. This twin can therefore quantitatively describe the three failure modes considered by the DT and thus prevent them. It operates as follows: if the phase level is too high, the rotational frequency is increased or a smaller weir is used. If the phase level is too low, the speed is reduced or a larger weir is used. If the system is limited by the coalescence, the DT can reduce the volume flow, which also increases the droplet size when using a static mixer and thus simplifies the coalescence. The rotational speed or phase ratio can also be changed online, which influences the coalescence in the rotor. These decisions can be made quickly and predictively using a DT, which means that such a failure mode does not have to be reached in order to react. This model-predictive control can be an advantage in the production of biologics. In addition, the DT can differentiate better between failure modes, since in production, the symptom of all failure modes is impure phases at the outlet. For example, if the HP leaves the extractor with LP impurities, this may be a coalescence limitation or a phase level that is too low. In the case of coalescence limitation, the simplest intervention would be to increase the rotational speed, but if the phase boundary level is too low, increasing the rotational speed would only lower it, and the problem would worsen.

By implementing the DT as a model-predictive control, the safe operating point for the space-time yield can be optimized, being maximal and reliably kept near the theoretical optimal operation point. Inaccuracies of the measuring devices and actuators are selected as safety margins in order to construct a worst-case scenario. As the accuracy of the devices used is very high with about less than 3–6%, a very optimized operating point can be achieved with the DT. Compared to the traditionally chosen safety factor of 30%, an increase in space-time yield of 25% can be achieved. In addition, the DT can support the design and predictive diagnosis of phase separation.

## Abbreviations

ACC: annular centrifugal contactor
ATPE: aqueous two-phase extraction
ATPS: aqueous two-phase system
CAPEX: capital expenditure
CQA: critical quality attributes
DT: digital twin
HP: heavy phase
LP: light phase
mRNA: messenger ribonucleic acid
PA: process attributes
pDNA: plasmid DNA
PEG: polyethylene glycol
QbD: Quality-by-Design
WCP: wet cell paste
